# Oral care medications for the prevention and treatment of ventilator-associated pneumonia in intensive care unit

**DOI:** 10.3389/froh.2025.1566355

**Published:** 2025-03-18

**Authors:** Hua Huang, Xiaomin Yu, Chenxi Huang, Jumei Zeng, Yuqing Li

**Affiliations:** ^1^ State Key Laboratory of Oral Diseases, National Center for Stomatology, National Clinical Research Center for Oral Diseases, West China Hospital of Stomatology, Sichuan University, Chengdu, China; ^2^ Department of Emergency Medicine, West China Hospital, Sichuan University/Nursing Key Laboratory of Sichuan Province, West China School of Nursing, Sichuan University, Chengdu, China; ^3^ West China-PUMC C.C. Chen Institute of Health, West China School of Public Health and West China Fourth Hospital, Sichuan University, Chengdu, China; ^4^ Center for Archaeological Science, Sichuan University, Chengdu, China

**Keywords:** ventilator-associated pneumonia, intensive care unit, oral medication and formulation, oral care, precise care

## Abstract

This study aims to ameliorate the management of VAP in clinical practice and deliver more precise care in the ICU. Study selection using the appropriate critical appraisal tools was undertaken by three authors. This review provides an overview of empirical antibiotics, chlorhexidine, and povidone-iodine, which are currently commonly used in critical care. It also discusses oral medications and preparations that may be used to prevent and treat ICU ventilator-associated pneumonia, including new antibiotics, hydrogen peroxide solutions, sodium bicarbonate, octenidine, and oral herbal medicines. It also discusses ongoing research and potential applications, such as the antimicrobial effects of these agents in ICU oral hygiene. Pharmaceuticals and formulations used in oral hygiene are effective or have huge application potential in the prevention and treatment of VAP, but further research is needed to standardize oral health assessment and care practices to develop evidence-based personalized oral hygiene for critically ill patients.

## 1 Introduction

**Figure 1:**
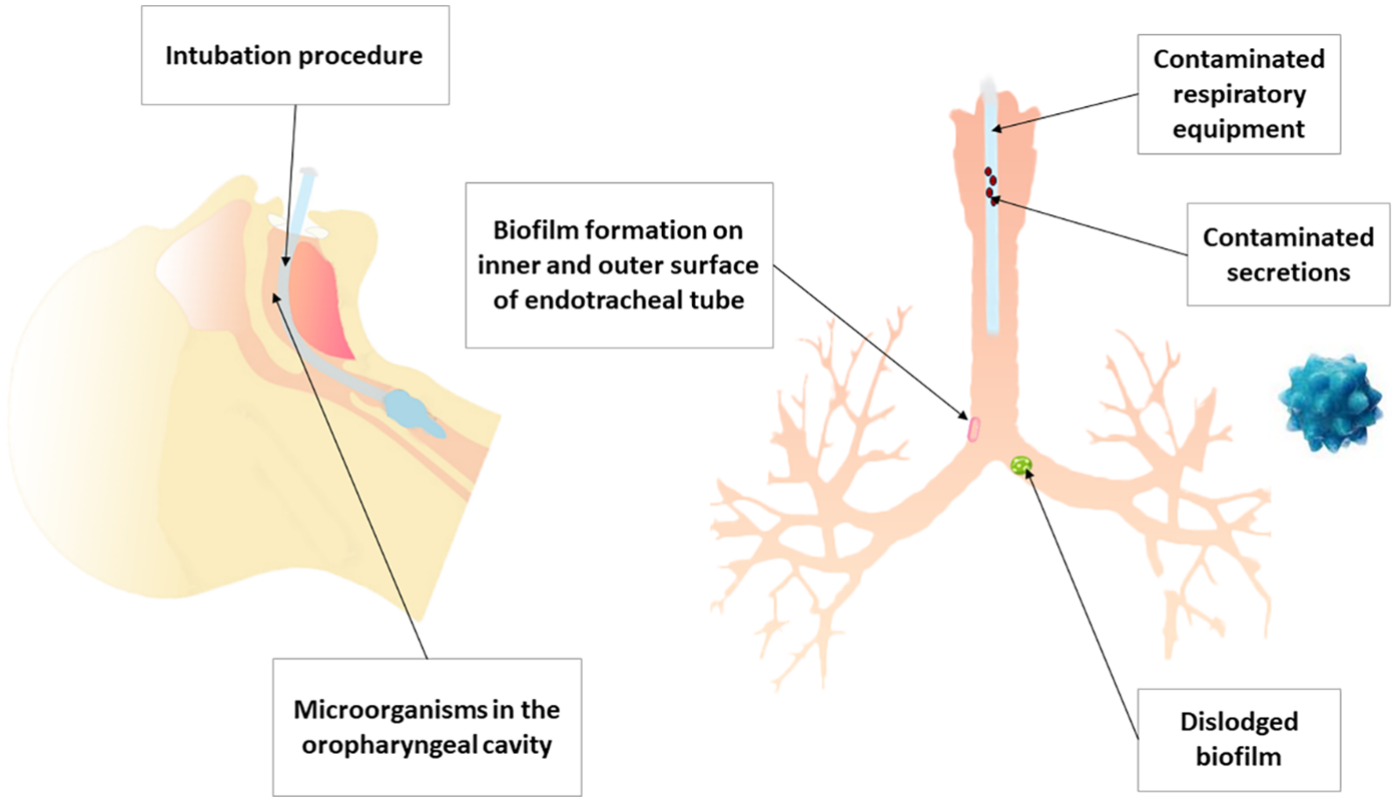
The pathogenic mechanism of VAP.

Ventilator-associated pneumonia (VAP) is a pulmonary parenchymal infection that develops in patients admitted to intensive care unit (ICU) and subjected to invasive mechanical ventilation for a minimum of forty eight hours (1). VAP is cause by a range of microorganisms affecting the respiratory system, which include Gram-negative bacterial such as *Klebsiella* spp., *Escherichia coli*, and *Pseudomonas aeruginosa*, as well as Gram-positive bacterial such as *Staphylococcus aureus*, including methicillin-resistant *S. aureus* (MRSA) (2). The advances in molecular diagnostic methods, which are deepening the comprehension of VAP microbiology, recent investigations discovered the presence of *Mycoplasma* in the bronchial lavage of patients diagnosed with VAP (3). Future therapeutic approaches will focus more on these microorganisms to prevent and treat VAP thanks to the deepening the understanding of the microbiome (4). The pathogenic mechanism of VAP is shown in Figure 1.

Endotracheal intubation represents a significant risk factor for VAP. VAP affects the body's airway defenses, hinders the ability to cough and clear mucus, and causes the accumulation of secretions containing bacteria above the inflatable cuff of the endotracheal tube that are aspirated (5), causing lower respiratory tract infections in ICU patients.

Previous studies indicated that dental plaque represents a significant reservoir for a variety of pathogens, since it contains a range of microorganisms associated with VAP (6). A study by Cindy L. Munro et al. (7) concluded that higher plaque scores increase the risk of VAP in more severely ill patients, especially those in the ICU. Therefore, the development of VAP may be reduced by a good and regular oral hygiene to prevent plaque accumulation and stimulate local oral immunity early in hospitalization (7, 8). Another study by Seok-Mo Heo et al. found that respiratory pathogens isolated from the lungs are often genetically indistinguishable from identical strains isolated from the oral cavity, suggesting that dental plaque is an important reservoir for respiratory pathogens in mechanically ventilated patients (9).

**Figure 2:**
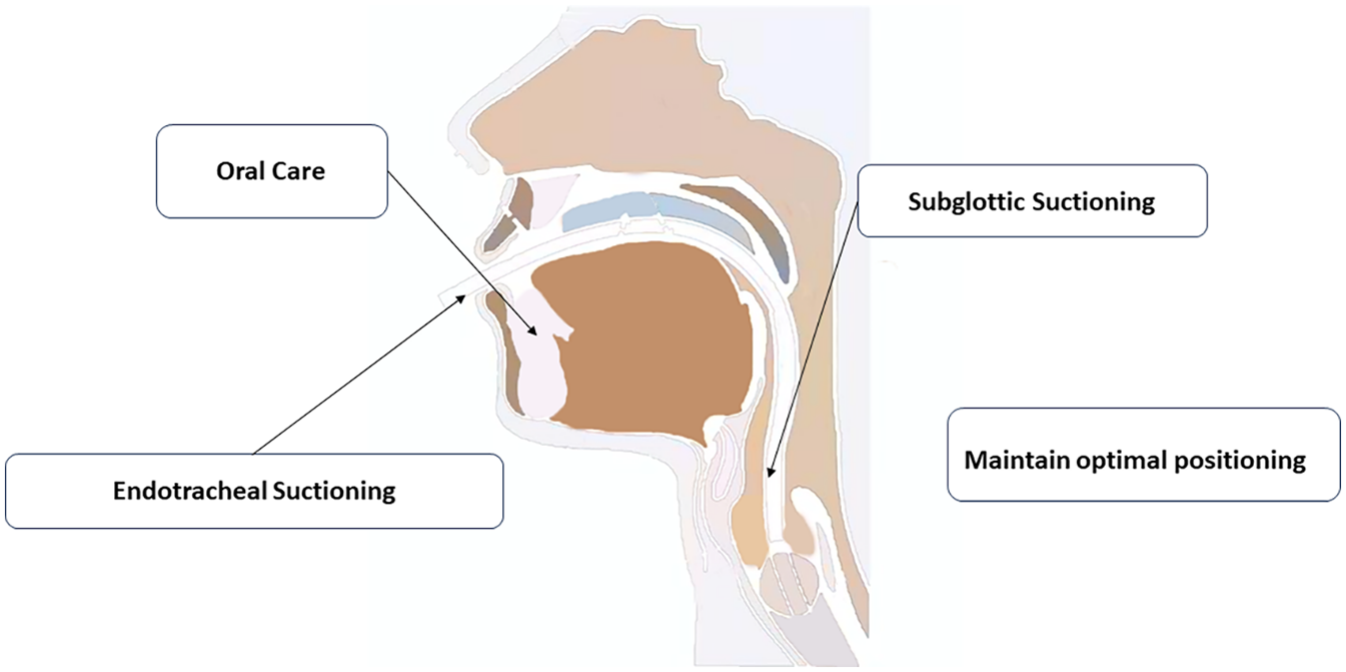
Various suggested methods to avoid VAP.

Numerous international organizations, such as the Centers for Disease Control and Prevention (CDC) and the American Association for Respiratory Care (AARC), promote the rigorous oral hygiene as a means of preventing VAP in patients undergoing mechanical ventilation (10). Figure 2 shows various suggested methods to avoid VAP.

Empirical antibiotics are the main drugs for preventing ventilator-associated pneumonia, but due to their potential side effects and the risk of antibiotic resistance, they need to be used with caution. Therefore, research on new drugs for ICU oral care is imminent.

Oral hygiene medications possess antimicrobial properties, thus used to prevent and treat infections of the oral cavity. Several antimicrobial agents commonly used in oral care encompass chlorhexidine and povidone iodine. This review provides a list of medications. It particularly emphasizes strategies for the effective prevention and management of oral issues in individuals who are unable to care for their oral hygiene and explores the potential application of drugs currently under research or development for oral hygiene in the ICU.

## 2 Methods

References for this review were identified through searches of PubMed for articles published from January, 1964, to April, 2024, by use of the terms “VAP”, “ICU care”, “oral care”, “oral medication and formulation”, “antibiotics”, “chlorhexidine”, “octenidine”, “povidone iodine”, “sodium bicarbonate” and “H_2_O_2_”. We reviewed guidelines for the management of VAP published by Centers for Disease Control and Prevention (CDC) and the American Association for Respiratory Care (AARC). We added articles through searches of the authors’ personal files. We also reviewed relevant references cited in retrieved articles. Articles published in English, Japanese and Portuguese were included. Pre-defined inclusion criteria were used.

## 3 Results and discussions

### 3.1 Antibiotics for VAP prevention

Antibiotic prophylaxis in VAP refers to the preventive administration of antibiotics to patients undergoing mechanical ventilation to reduce the incidence of this serious infection. The primary goal is to minimize the risk of VAP, particularly in high-risk populations, without significantly impacting mortality rates. Studies indicate that prophylactic antibiotics, especially when delivered via nebulization, can significantly lower VAP rates compared to control groups (11, 12). Compared with early empirical treatment, the use of prophylactic antibiotics is mainly aimed at reducing pathogen colonization and preventing the occurrence of VAP rather than treating existing infections (13).

Antibiotic prophylaxis in preventing VAP can be approached through systemic antibiotics, nebulized/inhaled antibiotics, and selective oropharyngeal decontamination (SOD). Each method has its commonly used agents and varying effectiveness. Systemic antibiotics, such as intravenous ampicillin-sulbactam, have shown efficacy in reducing VAP incidence, with a risk ratio (RR) of 0.62 in meta-analyses. However, these approaches do not significantly impact mortality rates or length of ICU stays (12). Nebulized antibiotics, particularly tobramycin and aminoglycosides, have demonstrated a significant reduction in VAP risk (RR of 0.69). The use of nebulized antibiotics was not associated with increased mortality or adverse events, making it a viable option for preventing VAP and the most interesting option for researchers (11, 14). SOD involves the use of topical antibiotics to reduce oropharyngeal colonization (15), though specific studies on its effectiveness compared to nebulized or systemic antibiotics are limited in the provided literature.

However, concerns about antimicrobial resistance (AMR) in the prevention of VAP are multifaceted, involving the need for effective infection control, judicious antibiotic use, and the development of preventive strategies. VAP is a significant concern in ICUs due to its association with high mortality rates and the frequent use of antibiotics, which can exacerbate AMR (16, 17). Long-term or extensive use of antibiotics, even when applied topically, may alter the local microbial ecology and selectively promote the growth of resistant strains, thereby increasing the risk of resistance. Its main mechanism includes antibiotic inactivation, that is, directly degrading the antibiotic or replacing the active group, destroying the structure of the antibiotic, thereby making the antibiotic lose its original function. Another mechanism is the cell efflux pump (18).

Besides, antibiotics induce adverse reactions such as diarrhea, nausea, vomiting, and allergic responses (19). While antibiotics are essential for reducing rates of VAP, their use needs careful consideration because of the above potential side effects and the risk of developing antibiotic resistance (20).

### 3.2 Oral care medications currently used in ICU

#### 3.2.1 Chlorhexidine

##### 3.2.1.1 Effects and benefits of chlorhexidine on VAP

Chlorhexidine is a commonly used antiseptic agent in diverse healthcare environments, including ICU. It is effective against a wide range of bacteria, both Gram-positive and Gram-negative linked to VAP (21); thus, it has been incorporated into oral hygiene regimens for ICU patients in hospitals worldwide (22). Several international organizations, such as the American Thoracic Society and the European Society of Intensive Care Medicine, support the use of chlorhexidine for oral hygiene in mechanically ventilated patients as a part of a complete treatment set for preventing VAP (23).

The administered oral chlorhexidine in the interventions varies in concentrations from 0.12% to 2%, and is provided in solution or gel form (24). A study among many on the comprehensive analysis of existing research regarding the influence of chlorhexidine on VAP revealed that 0.12% chlorhexidine is effective in preventing VAP in patients who subjected to cardiac surgery (24). Moreover, two studies by Koeman et al. and Hua et al. (21, 25), indicated that the oral administration of chlorhexidine from 0.12% to 2% reduces the incidence of VAP among a diverse group of critically ill patients, being used in various medical fields. Concurrently, a meta-analysis revealed a 37% decrease in the risk of VAP associated with the use of chlorhexidine (0.02%, 0.12%, 0.2%) (26).

##### 3.2.1.2 Resistance of bacteria isolated from the oral cavity to chlorhexidine

The development of resistance to antimicrobials is a significant and ongoing consequence in the use of chlorhexidine. A higher prevalence of chlorhexidine-resistant strains is present in the genera *Enterobacter*, *Pseudomonas*, *Proteus*, *Providencia*, and *Enterococcus*, especially *Enterococcus faecalis* (27). Chlorhexidine resistance is also present in multidrug-resistant strains, including *Klebsiella pneumoniae*. Several studies indicate that long-term exposure to chlorhexidine increases the probability of developing resistance to specific antibiotics (28, 29).

##### 3.2.1.3 Adverse effects of chlorhexidine

The predominant adverse effects associated with chlorhexidine mouthwash and gels are xerostomia, dysgeusia, alterations in tongue pigmentation, as well as calculus formation and extrinsic tooth discoloration after a prolonged use. Less frequently observed adverse effects include swelling of the parotid gland, sensation of a foreign body in the mouth, tongue pain, and desquamation of the oral mucosa (30). These effects are generally minor and reversible after stopping the use of this product (31). Chlorhexidine is banned in some countries due to cases of anaphylactic shock (32). Furthermore, an *in vitro* study demonstrated that chlorhexidine induces genotoxic and cytotoxic effects on human lymphocytes in a dose-dependent manner (33). Therefore, the risks of chlorhexidine should be taken into consideration to manage all relevant effects on the mouth.

#### 3.2.2 Povidone-iodine

Povidone-iodine is widely considered as one of the most effective antimicrobial agents in the reduction of the occurrence of respiratory infections, including VAP (34). It is effective against a wide spectrum of bacteria, including those frequently linked to VAP, and inhibits the development of biofilms on medical equipment (35).

In some countries, such as Japan, the use of chlorhexidine is restricted because of documented cases of anaphylactic shock; thus, povidone-iodine is preferred as an alternative substance (32). A randomized controlled study in Japan demonstrated that the topical administration of 10% povidone-iodine instead of 0.12% chlorhexidine for oral cleaning and irrigation effectively suppresses bacterial growth in the oropharyngeal fluid of mechanically ventilated patients without disturbing oral homeostasis (36). Another Japanese study found that the use of povidone-iodine mouthwash swabs, brushing teeth, and rinsing with 300 ml of acidic water reduces the risk of VAP in mechanically ventilated patients (37). A RCT study in France showed that the regular administration of povidone-iodine is an effective strategy to reduce the incidence of VAP in patients with traumatic brain injury (38).

However, a multicenter, randomized controlled trial did not find any evidence of the effect of povidone-iodine in preventing VAP; on the contrary, it increases the incidence of acute respiratory distress syndrome in patients with brain injury or cerebral hemorrhage (39). However, further research is recommended to investigate potential adverse effects considering the high possibility of bias in the included studies (40).

### 3.3 Other oral care medications with potential applications used in ICU

#### 3.3.1 Hydrogen peroxide

Hydrogen peroxide (H_2_O_2_) is a clear, colorless liquid with no odor. It is used in dentistry since the early 20th century, either alone or in conjunction with salts (41). The use of 3% H_2_O_2_ as a temporary oral debridement agent was approved by the FDA (42).

A study published in the Brazilian Journal of Infectious Diseases reported that individuals who used 3% H_2_O_2_ mouthwash twice daily experienced a 2.6-fold decrease in the risk of developing VAP in comparison to those who used saline mouthwash (43). Another study published in the journal of infection prevention reported that the incorporation of H_2_O_2_ mouthwash into a comprehensive VAP prevention system was linked to an evident decrease in VAP rates (44).

H_2_O_2_ also has some side effects. Of particular concern are the reports on the genotoxic effects of H_2_O_2_ in bacteria and mammalian cells. The prolonged use of 3% H_2_O_2_ in animal experiments results in a slight increase in the formation of precancerous lesions and tumors (45). Additional research is needed to confirm the long-term safety and effectiveness of H_2_O_2_ in the prevention of VAP. It is recommended to use caution when using it and to incorporate it as part of a comprehensive strategy to prevent VAP.

#### 3.3.2 Sodium bicarbonate

Sodium bicarbonate, commonly referred to as baking soda, has various uses in the oral cavity. It is known for its safety, minimal abrasiveness, and bactericidal properties, making it a patient-friendly option for mouthwash, and as a component in toothpaste and chewing gum. It can be used as a long-term treatment as an adjunct with virtually no side effects (46). As a result, sodium bicarbonate has a significant potential for application in the oral hygiene in the ICU to prevent VAP.

Sodium bicarbonate possesses bactericidal and neutralizing properties, making its use suitable as a mouthwash (47), decreasing the quantity of bacteria in the oral cavity. This action disrupts the adherence of bacteria to the tooth, consequently reducing plaque formation.

Toothpaste containing sodium bicarbonate possesses superior bactericidal activity compared to other toothpastes due to its safety, low cost, low abrasiveness, water solubility, buffering capacity, and compatibility with fluoride (48). Toothpastes containing sodium bicarbonate are effective in removing plaque and higher concentrations of sodium bicarbonate are associated with greater efficacy (49, 50). Another study indicated that baking soda toothpaste is more effective than those without baking soda. Antibacterial toothpaste is effective in removing plaque after just one brushing session and maintain significantly lower plaque levels during four weeks of twice-daily and unsupervised brushing (51). Toothpaste containing sodium bicarbonate effectively removes plaque before it irreversibly binds to the teeth, thus ensuring an environment for good oral health (52).

Nevertheless, individuals with hypertension and those adhering to a low-sodium diet should be careful when considering the use of sodium bicarbonate mouthwash (50). Further research is necessary to evaluate the concentration and duration needed for its bactericidal effect without causing harmful effects on the oral mucosa.

#### 3.3.3 Octenidine

Octenidine(OCT) has lower systemic toxicity than chlorhexidine (53, 54), which may be due to the lack of amide and ester structures in its molecule (55). It has a broad range of antimicrobial activity, affecting Gram-positive bacteria, Gram-negative bacteria, Chlamydia, Mycoplasma and fungi (56).

A study indicated that 0.1% OCT is an effective antiplaque agent. Compared with placebo, octinoxate inhibited plaque formation by up to 93%, which is comparable to chlorhexidine (57). Another study showed that OCT 0.1% mouthwash inhibits plaque formation for more than 5 days. Therefore, it can be recommended when regular oral hygiene is temporarily impaired (58). Moreover, OCT can inhibit the colonization of Staphylococcus aureus and Pseudomonas aeruginosa on tracheotomy tubes and reduce the colonization and biofilm formation on the surface of polymer tracheotomy tubes, which has potential application value in the prevention and treatment of VAP (59).

Although the safety of OCT has been confirmed by research, potential risks still exist. The use of OCT solution may penetrate wounds or skin and cause chronic inflammation (60, 61). However, these findings have limitations and need to be verified by more precise studies.

## 4 Advice and recommendation for future studies on VAP oral care

To improve patient outcomes through continued research into ventilator-associated pneumonia, attention should be focused on the development and improvement of diagnostic methods and therapeutic drugs. More specific diagnostic methods are needed to select the appropriate antimicrobial drugs for specific pathogens. It is also crucial to further study the mechanisms of antibiotic resistance and specific characteristics of oral microorganisms. This could help address multi-drug resistance of oral microorganisms, thus improving the effectiveness of oral medications in the treatment of VAP.

In terms of diagnosis, point-of-care (POCT) tools are diagnostic tests that can be used at or near the bedside, with delays ranging from minutes to hours. The use of POCTs for the diagnosis of VAP allows for faster diagnosis and adjustment of antimicrobial therapy. New testing methods such as multiplex polymerase chain reaction (MPCR), exhaled breath group analysis, and chromogenic tests have also been developed (62).

In terms of therapy, multidrug-resistant bacteria are a growing concern in the medical field. According to a recent article published in Frontiers in Bioengineering and Biotechnology, nanocarrier systems can be used to design and develop efficient antimicrobials for multidrug-resistant bacteria (63). These nano-sized antimicrobials have a predominance over traditional antibiotics because their small size helps them in better interaction with bacterial cells. Moreover, surface engineering of nanocarriers offers significant advantages of targeting and modulating various resistance mechanisms, thus owe superior qualities for overcoming bacterial resistance (63).

However, the development of new drugs is a complex process that requires extensive research and testing. It is important to note that the development of new drugs is highly regulated by government agencies such as the FDA in the United States. The process typically involves several stages, including preclinical testing, clinical trials, and regulatory review (8).

In terms of developing new drugs to treat multidrug-resistant bacteria based on the multidrug resistance of oral microorganisms, it is important to conduct extensive research on the mechanisms of antibiotic resistance and the specific characteristics of oral microorganisms. This research can help identify potential targets for drug development and inform the design of new drugs (63).

## 5 Conclusion

The contribution of oral bacteria to the development of pneumonia is well known. However, there is controversy regarding best practices for achieving optimal oral health care in the context of critical care. Drugs and formulations used in oral hygiene are effective in preventing VAP, but they still have inherent drawbacks. Studies of some other drugs have demonstrated their promise in ICU oral care instead of empirical antibiotics, and the development of new drugs is also worth looking forward to, but there is still a long way to go to transfer microbiology and drug research from the laboratory to the clinic, and promising technologies require more research before they become widely available. Further research is needed to standardize oral health assessment and care practices to develop evidence-based personalized oral hygiene for critically ill patients. Our hope is that this review might serve as a useful reference for ICU oral care and the prevention of VAP.
